# Clinical Effectiveness of Treatments for Mild Cognitive Impairment in Adults: A Systematic Review

**DOI:** 10.3390/ejihpe15110226

**Published:** 2025-11-03

**Authors:** Daniel Cepeda-Pineda, Gabriela Sequeda, Sandra-Milena Carrillo-Sierra, Kevin Silvera-Cruz, Johanna Redondo-Chamorro, Astrid Rozo-Sánchez, Valmore Bermúdez, Julio César Contreras-Velásquez, Yulineth Gómez-Charris, Diego Rivera-Porras

**Affiliations:** 1 Universidad Simón Bolívar, Facultad de Ciencias Jurídicas y Sociales, Cúcuta 540001, Colombia; d_cepeda@unisimon.edu.co; 2 Facultad de Ciencias del Comportamiento, Universidad de la Sabana, Chía, Cundinamarca 111321, Colombia; 3 Grupo de Investigación en Modelamiento Científico e Innovación Empresarial, Facultad de Ciencias Jurídicas y Sociales, Universidad Simón Bolívar, Cúcuta 540001, Colombia; zuly.sequeda@unisimon.edu.co; 4 Universidad Simón Bolívar, Facultad de Ciencias Jurídicas y Sociales, Centro de Investigación en Estudios Fronterizos, Cúcuta 540001, Colombia; 5 Universidad Simón Bolívar, Facultad de Ciencias de la salud, Barranquilla 080001, Atlántico, Colombia; kevin.silvera@unisimon.edu.co; 6 Universidad Popular del Cesar, Departamento de Psicología, Valledupar 200001, Colombia; johanna-redondo@unicesar.edu.co; 7 Universidad de Pamplona, Facultad de Salud, Pamplona 543050, Colombia; astrid.rozo@unipamplona.edu.co; 8 Universidad Simón Bolívar, Centro de Investigaciones en Ciencias de la Vida, Barranquilla 080001, Colombia; valmore.bermudez@unisimon.edu.co; 9 Universidad de la Costa, Departamento de Productividad e Innovación, Barranquilla 080001, Atlántico, Colombia; jcontrer30@cuc.edu.co (J.C.C.-V.); ygomez6@cuc.edu.co (Y.G.-C.)

**Keywords:** mild cognitive impairment, drug therapy, non-pharmacologic therapy, cognition, treatment outcome

## Abstract

**Background/Objectives**: Mild cognitive impairment (MCI) represents an intermediate stage between normal ageing and dementia, with a high annual progression rate. Despite its clinical relevance, no pharmacological treatment has been definitively approved for this condition; however, multiple pharmacological and non-pharmacological strategies have been investigated for their potential benefits. This systematic review assessed the effectiveness of both types of interventions in adults with MCI, aiming to identify effective strategies to preserve cognitive function. **Methods**: A systematic search (2017–2025) was conducted in PubMed, Scopus, ScienceDirect, SpringerLink, and WOS, following PRISMA guidelines. Randomised controlled trials and quasi-experimental studies involving adults aged ≥ 50 years with a diagnosis of MCI were included. Outcomes were evaluated in terms of cognitive, functional, behavioural, and quality-of-life improvements. Risk of bias was assessed using the RoB 2 and ROBINS-I tools. **Results**: Of 108,700 records screened, 40 studies were included. Non-pharmacological interventions, such as cognitive training (conventional, computerised, or virtual reality-based), consistently improved memory, attention, and executive functions (e.g., MoCA: +3.84 points; *p* < 0.001). Transcranial magnetic stimulation combined with physical exercise also demonstrated significant benefits (*p* = 0.025). Among pharmacological treatments, only vortioxetine and choline alfoscerate showed modest improvements; cholinesterase inhibitors had limited effects and frequent adverse events. Complementary therapies (yoga, probiotics, and acupuncture) yielded promising outcomes but require further validation. **Conclusions**: Non-pharmacological strategies, particularly cognitive training and physical exercise, emerge as the most effective and safe approaches for managing MCI. The inclusion of pharmacological interventions with preliminary evidence of benefit should be considered within a personalised, multimodal approach, while recognising the current absence of approved drug treatments for MCI. Further research is needed in underrepresented populations, such as those in Latin America.

## 1. Introduction

Mild Cognitive Impairment (MCI) constitutes an intermediate clinical stage, a diagnostic crossroads between the cognitive changes associated with normal ageing and the early manifestations of overt dementia (Feldman & Jacova, 2005). It is defined as a syndrome characterised by an objective decline in one or more cognitive functions—such as memory, attention, or language—that, although noticeable to the individual or those around them, does not significantly impair functional independence in daily life (Knopman & Petersen, 2014). Owing to its clinical and aetiological heterogeneity, MCI continues to pose a challenge to the development of a universal definition and a standardised diagnostic profile (Hughes et al., 2011).

Although current classification systems such as the DSM-5 and ICD-11 favour an aetiological approach and avoid the term “impairment”, Ronald Petersen’s proposal from the late 1990s remains a cornerstone in clinical practice. His pragmatic model suggests subtypes of MCI as potential precursors of various dementia syndromes. This classification comprises four categories (González Martínez et al., 2021): (a) Amnestic single-domain MCI: exclusively affects memory. (b) Amnestic multiple-domain MCI: involves memory and other cognitive domains. (c) Non-amnestic single-domain MCI: affects a non-memory cognitive function, such as language or executive functioning. (d) Non-amnestic multiple-domain MCI: involves several cognitive functions with relatively preserved memory.

The continued relevance of this typology lies in its clinical utility: it facilitates the early identification of individuals at high risk of progressing to dementia. Within this framework, the concept of subjective cognitive decline has emerged, referring to an individual’s perception of declining cognitive ability, often preceding objective test detection (Pavel et al., 2023). However, a reliable diagnosis of MCI requires a comprehensive neuropsychological assessment. A cognitive performance at least 1.5 standard deviations below the normative mean—adjusted for age and educational level—is typically considered indicative of impairment (Jak et al., 2009).

In clinical settings, recurrent memory lapses are often the first warning sign. A longitudinal study of 148 participants found that, in 118 of them, memory failures were the initial symptom, later accompanied by difficulties in daily activities and temporal-spatial disorientation (Devier et al., 2010). However, MCI is not purely a cognitive phenomenon. A systematic review and meta-analysis by (John et al., 2019) revealed that affective symptoms such as depression and anxiety are common in MCI and are associated with a 36% increased risk of developing the condition (OR = 1.36; 95% CI: 1.05–1.76; *p* = 0.02). Behavioural symptoms such as irritability, agitation, and aggression have also been linked to faster cognitive decline (Bidzan et al., 2023).

Age remains the most significant non-modifiable risk factor for MCI. Although onset varies, studies commonly report its emergence between the ages of 60 and 66 (Moustaka et al., 2023). A study by (Molano et al., 2010) in the United States found that approximately two in every three older adults exhibit some degree of cognitive impairment by the age of 70 (Hale et al., 2020). Additional risk factors—including low educational attainment, tobacco use, and alcohol or substance consumption—have been consistently associated with increased MCI risk (Muhammad et al., 2021; Ware et al., 2024). Furthermore, genetic factors—most notably the APOE ε4 allele—represent a well-established non-modifiable risk. APOE ε4 carriers show elevated risk of MCI and Alzheimer’s disease, with one allele increasing risk two- to three-fold and homozygosity conferring up to a ~15-fold increase, as well as earlier age at onset; APOE ε4 is also associated with greater hippocampal atrophy in individuals with MCI (Hu et al., 2017; Jefferson et al., 2015; C.-C. Liu et al., 2013; Petersen et al., 2010). Gender, on the other hand, remains a debated factor with no clear consensus in the literature (Y. Liu et al., 2022). Notably, while major depression is often linked to MCI, some reviews suggest it may be more of an early consequence of emerging brain changes than a predictor or biomarker of the condition (González Hernández et al., 2022).

From an epidemiological perspective, MCI represents a growing public health concern. A systematic review by Petersen estimated that global prevalence among adults over the age of 60 ranges between 12% and 18%. Even more concerning is the annual conversion rate: between 8% and 15% of individuals with MCI progress to dementia each year (Petersen, 2016). In Colombia, a recent situational analysis involving 23,694 participants reported a prevalence of cognitive impairment without dementia of 8.9%, underscoring its local significance (Guerrero Barragán et al., 2023).

In response to this scenario, therapeutic interventions are generally divided into two main approaches: pharmacological and non-pharmacological. The former, involving cholinesterase inhibitors (also used in Alzheimer’s disease), faces a major limitation: to date, no medication has been approved by the US Food and Drug Administration (FDA) specifically for the treatment of MCI (Andrango Pilataxi & López Barba, 2022; Chertkow et al., 2008). Multiple meta-analyses, including those by (Fitzpatrick-Lewis et al., 2015) and (Raschetti et al., 2007), have failed to demonstrate significant cognitive benefits over placebo, and instead report a higher incidence of adverse effects.

Given the limited effectiveness of pharmacological treatments, non-pharmacological strategies have gained increasing attention. Cognitive stimulation has emerged as the most robust intervention, supported by a substantial body of evidence indicating its capacity to maintain, improve, and particularly slow the progression of cognitive decline (Carcelén-Fraile et al., 2022). Additional approaches—such as transcranial magnetic stimulation (Malavera et al., 2014), regular physical exercise (Russo et al., 2020), and nutritional interventions (Buckinx & Aubertin-Leheudre, 2021; Power et al., 2020)—have also shown promising results. Meanwhile, psychotherapy has proven to be a relevant tool, particularly in light of the frequent comorbidity of MCI with mood disorders or impulse control difficulties (Apostolova et al., 2014; Ginsberg et al., 2019; Saari et al., 2021). It contributes not only to emotional well-being but may also positively influence the functional and behavioural course of the condition.

In this context, the present systematic review seeks to critically examine the therapeutic interventions available for MCI, assessing their reported effectiveness and real-world applicability. The significance of this study is twofold: on one hand, it addresses the growing scientific interest in this clinical phenomenon; on the other, it aims to identify the most effective strategies to potentially alter the course of a condition that frequently precedes devastating neurodegenerative diseases such as dementia (Katsuno et al., 2018).

## 2. Materials and Methods

This systematic review was conducted following the PRISMA guidelines (Page et al., 2021), with the aim of identifying, analysing, and synthesising the best available evidence on the various types of therapeutic interventions—both pharmacological and non-pharmacological—directed at individuals formally diagnosed with MCI, aged 50 years and older. The analysis focused not only on the primary and secondary effects of these interventions, but also on the methodological quality of the findings reported. The review protocol is available in the International Prospective Registry of Systematic Reviews (PROSPERO) with the code number PROSPERO 2025 CRD420251121047, version 1.1, published 6 August 2025.

### 2.1. Research Question

The guiding question for this review was structured using the PICO model, which facilitates the clear organisation of the central components of a clinical review. In community-dwelling adults (≥50 years) with MCI (P), do pharmacological or non-pharmacological interventions (I), compared to placebo or standard care (C), improve cognition, functionality, behaviour, general health status, or mortality (O)? (see Table 1).

### 2.2. Inclusion Criteria

To ensure the relevance and methodological quality of the studies selected, specific inclusion criteria were established. Eligible studies were those published between 2017 and 2025, in order to guarantee the timeliness and applicability of the evidence. The studies had to include participants aged 50 years or older, with a formal diagnosis of MCI based on recognised clinical criteria, specifically the Petersen criteria or the National Institute on Aging–Alzheimer’s Association (NIA-AA) guidelines. All included studies adhered to one of these two diagnostic frameworks; no other diagnostic criteria were accepted. For cognitive screening, studies were required to report acceptable cut-off scores, defined as a Montreal Cognitive Assessment (MoCA) score ≤ 25 or a Mini-Mental State Examination (MMSE) score ≤ 27, ensuring consistency in participant selection across studies. These thresholds were applied as part of our own screening process during eligibility assessment, in addition to verifying that the original studies reported values within these ranges. Only articles written in English were included, due to considerations related to accessibility, standardisation, and critical appraisal. Furthermore, only studies with experimental or quasi-experimental designs were selected, as these provide greater rigour in assessing the effectiveness of interventions. Lastly, the studies were required to explicitly examine pharmacological or non-pharmacological interventions targeting MCI, with an evaluation of their effects on clinical, cognitive, or functional outcomes.

### 2.3. Exclusion Criteria

Studies that did not meet the aforementioned criteria were excluded from the review. In particular, research published prior to 2017 or involving participants under the age of 50 was not considered. Studies that did not focus primarily on MCI—such as those addressing subjective cognitive decline, dementia, or other neurocognitive disorders—were also excluded, as were publications in languages other than English. Similarly, studies employing descriptive designs, systematic reviews, or narrative reviews were excluded, as they do not allow for direct evaluation of intervention outcomes. Finally, any study that failed to clearly assess either pharmacological or non-pharmacological therapeutic approaches to MCI was excluded from the analysis.

#### Search Strategy

The search strategy was carefully designed to maximise both sensitivity and specificity in identifying relevant scientific literature on therapeutic interventions for MCI. Key terms commonly used in the fields of clinical neuroscience and geriatrics were selected and adapted into English using standardised descriptors from DeCS (Health Sciences Descriptors) and MeSH (Medical Subject Headings). This allowed the construction of robust search algorithms that incorporated synonyms and related terms, thereby enhancing thematic coverage without compromising precision (see Table 2).

### 2.4. Databases and Search Algorithms

Based on the selected terms, combined search algorithms were developed using Boolean operators (AND, OR, NOT) and applied across the following databases: PubMed, Scopus, Web of Science, ScienceDirect, and SpringerLink. These databases were chosen for their broad thematic coverage and relevance to fields such as mental health, geriatrics, and neuropsychology. The search process was conducted between November 2024 and April 2025 by the research team. Each algorithm was adapted to the specific requirements of each database, which helped to optimise article retrieval and minimise the risk of omitting relevant records (see Table 3).

### 2.5. Database-Specific Application

Table 4 presents the distribution of search algorithms across the different databases. Each team was assigned a specific combination and was responsible for adapting the algorithms to the maximum or minimum number of terms allowed by each platform. This ensured rigorous control over the search process and enhanced the replicability of the methodology.

### 2.6. Data Collection

The information was organised and systematised using a state-of-the-art matrix developed in Microsoft Excel, complemented by a compilation table that enabled the identification, recording, and documentation of the metadata of the studies selected for the final sample. Data extraction included key information such as sample size, characteristics of the intervention (treatment applied to the experimental group), study duration, and the main findings reported.

## 3. Results

This section presents the selected studies and the findings related to interventions for MCI, following a rigorous process of search, selection, and analysis of the studies included in the systematic review. Based on a carefully planned methodological strategy, relevant evidence was identified that addresses the objectives of the research, ensuring transparency and traceability throughout the entire process.

### 3.1. Study Selection

**Figure 1:**
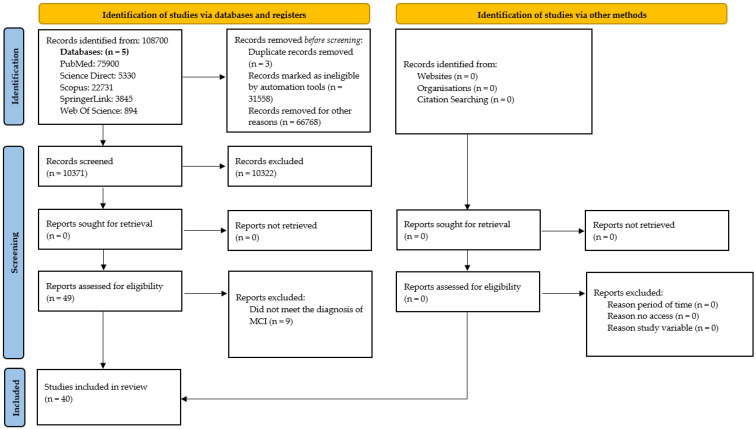
PRISMA Flow Diagram. Source: adapted from PRISMA 2020 guidelines (Page et al., 2021). Note: The flow diagram outlines the study selection process in accordance with the PRISMA guidelines. From an initial 105,700 records identified across the five databases consulted, 40 met all the inclusion criteria and were incorporated into the review. The exclusion of 9 studies during the eligibility assessment phase was primarily due to non-adherence to the established diagnostic criteria for Mild Cognitive Impairment (MCI). No additional studies were added through manual searches or other sources.

The PRISMA flow diagram (see Figure 1) illustrates the process of study selection undertaken in this systematic review (Rethlefsen & Page, 2021), covering the phases of identification, screening, eligibility assessment, and inclusion. It also outlines the reasons for the exclusion of studies that did not meet the established criteria.

### 3.2. Risk of Bias Analysis

The risk of bias assessment was conducted using Cochrane’s RoB 2 tool for a total of 37 randomised controlled trials (RCTs). As for the remaining three studies, which did not meet the methodological criteria of a controlled clinical trial, the ROBINS-I tool was employed in order to rigorously evaluate their methodological quality and responsibly integrate their evidence into this review.

**Figure 2:**
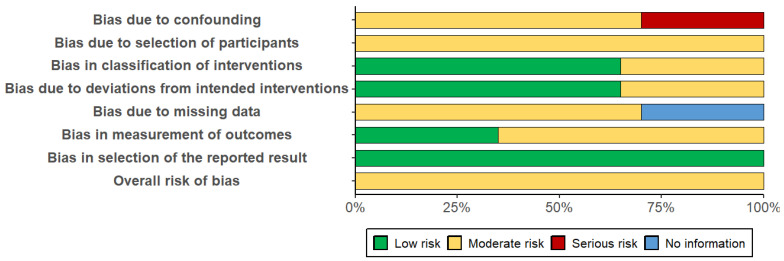
Percentage distribution of risk of bias. Source: Own elaboration based on the ROBINS-I tool. Note: The figure shows the percentage of studies classified within each domain of the ROBINS-I tool. The colours represent the level of risk of bias: green indicates low risk of bias, yellow indicates moderate risk of bias, red indicates serious risk of bias, and blue indicates insufficient information. The assessed domains include: bias due to confounding, bias due to selection of participants, bias in classification of interventions, bias due to deviations from intended interventions, bias due to missing data, bias in measurement of outcomes, and bias in selection of the reported result. The category Overall risk of bias reflects the overall risk of bias level across studies.

**Figure 3:**
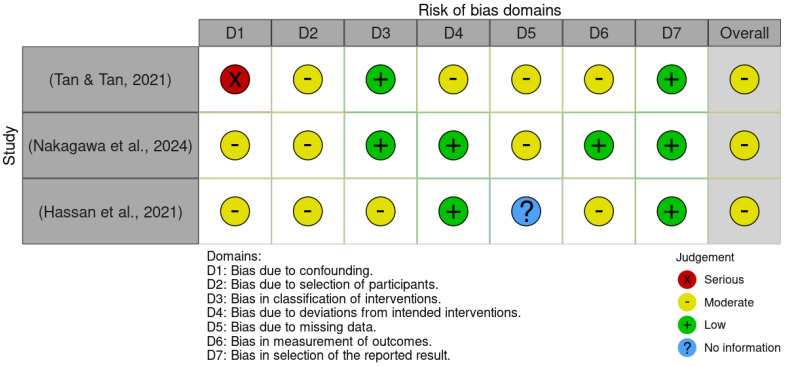
Risk of bias assessment by domain. Source: Own elaboration based on the ROBINS-I tool (Tan & Tan, 2021; Nakagawa et al., 2024; Hassan et al., 2021). Note: The figure shows the risk of bias diagram (ROBINS-I) applied to quasi-experimental clinical trials. The colours represent the bias judgement for each domain: green denotes low risk of bias, yellow denotes moderate risk of bias, and red denotes serious risk of bias. The evaluated domains are: D1 (bias due to confounding), D2 (bias due to selection of participants), D3 (bias in classification of interventions), D4 (bias due to deviations from intended interventions), D5 (bias due to missing data), D6 (bias in measurement of outcomes), D7 (bias in selection of the reported result), and “overall” indicates the overall risk of bias for each study.

Figure 2 and Figure 3 present the risk of bias evaluation for the non-randomised studies. Although the primary focus of this systematic review was on randomised controlled trials, the inclusion of these three studies was considered appropriate, as they examined potentially relevant interventions in the context of pharmacological and combined treatment approaches. Their inclusion broadened the spectrum of available evidence, particularly regarding the use of medication, since most experimental studies tend to focus on dementia interventions, and few address MCI.

As expected, the three non-randomised studies displayed methodological limitations inherent to their design. The most affected domains were: confounding (D1), selection of participants (D2), and handling of missing data (D5). Issues identified included the lack of comparison groups, unclear participant selection procedures, and inadequate (or unreported) management of attrition and missing data.

Nevertheless, the domain related to selective reporting (D7) showed no signs of bias in any of the three studies, suggesting that outcomes were reported completely and without manipulation. Additionally, the domains concerning intervention during the study (D3) and deviations from intended intervention (D4) did not reveal significant bias, indicating that the intervention was administered appropriately and without major deviations.

Following the comprehensive evaluation, it was concluded that the three studies presented an overall moderate risk of bias. Accordingly, their findings should be interpreted with caution, close attention to detail, and avoidance of unwarranted generalisations. However, we argue that these findings should not be disregarded, as they offer valuable insights into interventions that are seldom explored in research on MCI, particularly within the pharmacological domain.

**Figure 4:**
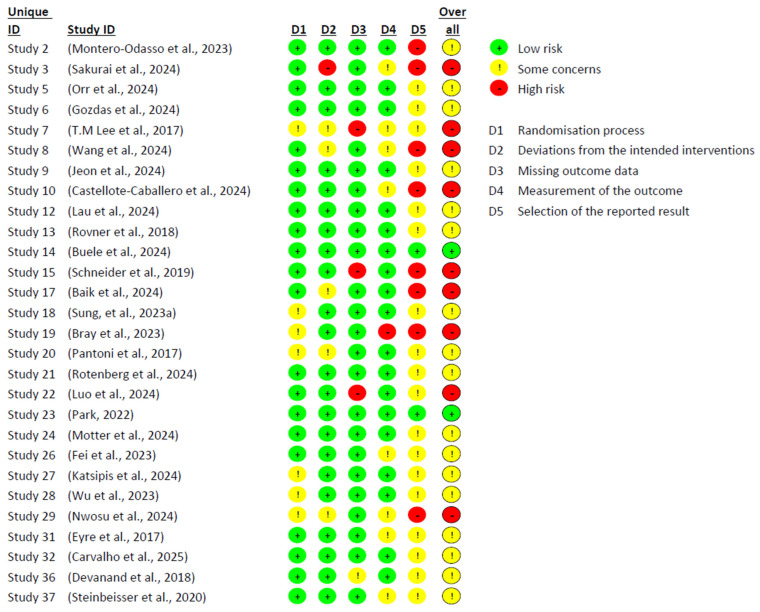
Risk of bias assessment by domain, intention to treat. Source: Own elaboration based on the Cochrane’s RoB 2 tool (Montero-Odasso et al., 2023; Sakurai et al., 2024; Orr et al., 2024; Gozdas et al., 2024; T. M. Lee et al., 2017; Wang et al., 2024; Jeon et al., 2024; Castellote-Caballero et al., 2024; Lau et al., 2024; Rovner et al., 2018; Buele et al., 2024; Schneider et al., 2019; Baik et al., 2024; Sung et al., 2023a; Bray et al., 2023; Pantoni et al., 2017; Rotenberg et al., 2024; Luo et al., 2024; Park, 2022; Motter et al., 2024; Fei et al., 2023; Katsipis et al., 2024; Wu et al., 2023; Nwosu et al., 2024; Eyre et al., 2017; Carvalho et al., 2025; Devanand et al., 2018; Steinbeisser et al., 2020). Note: In the bias potential diagram (RoB 2), the colours indicate the degree of bias potential for each area assessed: green represents low bias potential (no bias), yellow represents some risk (moderate bias potential) and red represents high bias potential. Columns D1, D2, D3, D4, D5 and ‘overall’ correspond to the different areas of bias potential assessed using the RoB 2 tool, including the overall assessment of bias potential for the study.

**Figure 5:**
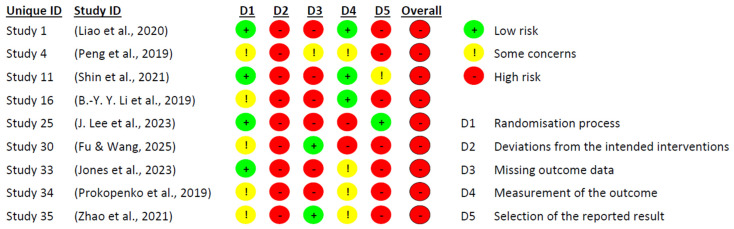
Risk of bias assessment by domain, per protocol. Source: Own elaboration based on the Cochrane’s RoB 2 tool (Liao et al., 2020; Peng et al., 2019; Shin et al., 2021; B.-Y. Y. Li et al., 2019; J. Lee et al., 2023; Fu & Wang, 2025; Jones et al., 2023; Prokopenko et al., 2019; Zhao et al., 2021). Note: In the risk of bias (RoB 2) diagram, the colours indicate the level of risk of bias for each assessed domain: green denotes low risk of bias (free of bias), yellow denotes some concerns (moderate risk of bias), and red denotes high risk of bias. The columns D1, D2, D3, D4, D5 and “overall” correspond to the different risk of bias domains assessed by the RoB 2 tool, including the overall judgement of risk of bias for the study. This figure presents only those trials that performed a per-protocol analysis.

Figure 4 and Figure 5 present the risk of bias analysis, conducted using Cochrane’s RoB 2 tool, which enabled an assessment of the methodological quality of the 37 included studies. Significant limitations were identified in the design and implementation of the trials, which affected the reliability of the findings and reduced their internal validity. Only 2 studies were classified as having a low risk of bias across all domains, highlighting the persistent methodological challenges in research on MCI. One of the most critical sources of bias was the type of statistical analysis employed. While 28 studies used an intention-to-treat approach, 9 conducted per-protocol analyses. The latter were generally associated with a higher risk of bias, likely due to the exclusion of participants who did not complete the intervention, potentially introducing systematic distortions in the outcomes.

Domain 5—related to outcome reporting—was the most frequently compromised. In many studies, pre-specified protocols were either not declared or not accessible, making it difficult to compare planned and reported outcomes. Furthermore, signs of post hoc decisions and unreported exploratory analyses were identified, which may have artificially inflated the observed effects. In contrast, Domains 1 (random sequence generation), 2 (deviations from intended interventions), and 3 (missing outcome data) showed comparatively lower risk, though not without issues. Instances such as lack of allocation concealment, absence of blinding among intervention staff, participant attrition, and poor adherence to protocols were still observed. Many of these limitations appeared to be closely linked to the nature of non-pharmacological interventions—such as cognitive training, physical exercise, or psychosocial programmes—in which blinding is often unfeasible and adherence tends to fluctuate considerably.

### 3.3. Characteristics of the Included Studies

Following a rigorous and systematic literature review, 40 studies were identified that met the established eligibility criteria. The majority of these (n = 37) were randomised controlled trials (RCTs), while two were single-arm studies and one employed a quasi-experimental design. Table 5 provides a summary of the main methodological characteristics and the most relevant findings reported in each of the included studies.

The studies included in this review display a diverse geographical distribution, with a predominance in countries across Asia, North America, and Europe. Latin America, by contrast, was underrepresented, highlighting a significant research gap in the region. China, South Korea, the United States, and Canada (Jeon et al., 2024; Montero-Odasso et al., 2023; Peng et al., 2019; Rovner et al., 2018) accounted for the highest concentration of studies, which limits the generalisability of findings and underscores the need to strengthen scientific output on MCI in Latin American contexts.

Regarding methodological approaches, most studies employed clinical trials as their primary design, in accordance with the inclusion criteria established. However, only a small number of studies adopted alternative designs, such as single-arm or quasi-experimental approaches (Hassan et al., 2021; Nakagawa et al., 2024). This indicates a predominance of controlled investigations, while also suggesting the need to broaden methodological perspectives in order to better capture the complexity of the phenomenon.

The duration of interventions varied considerably, ranging from 4 to 156 weeks. Nevertheless, the majority of trials were concentrated within periods of 8 to 26 weeks, suggesting that short- to medium-term interventions may be sufficient to produce cognitive improvements (Buele et al., 2024; Jones et al., 2023; Lau et al., 2024). This observation reinforces the value of implementing accessible and sustainable programmes, particularly in settings with limited resources.

In terms of therapeutic strategies, a wide variety of non-pharmacological treatments were identified. Cognitive training was the most frequently used intervention and also reported the most favourable outcomes in terms of cognitive function enhancement (Gozdas et al., 2024; Sung et al., 2023b), a finding that aligns with the specialised literature. Additional interventions such as transcranial magnetic stimulation, yoga, the use of probiotics, and the administration of vortioxetine were also evaluated. These showed promising effects by targeting biological mechanisms related to oxidative stress, brain inflammation, and neural connectivity (Eyre et al., 2017; Fei et al., 2023; Lau et al., 2024; Tan & Tan, 2021).

Sample sizes varied considerably across studies: while some trials involved small groups (20 to 50 participants), others engaged much larger populations (Sakurai et al., 2024; Steinbeisser et al., 2020). Despite this variability, most investigations reported statistically significant improvements following the intervention, particularly in domains such as memory, attention, processing speed, and verbal fluency. However, not all studies yielded positive results; notably, some trials involving pharmacological interventions—such as ladostigil, donepezil, and nicotinamide riboside—did not demonstrate significant effects compared with control or placebo groups (Devanand et al., 2018; Orr et al., 2024; Schneider et al., 2019).

Multimodal interventions based on lifestyle changes also emerged as a particularly relevant strategy. The combination of physical activity, healthy nutrition, and sleep hygiene not only showed cognitive benefits but also supported a holistic approach to wellbeing, with potential for both preventive and therapeutic applications (Wang et al., 2024).

Finally, the criteria used to diagnose MCI varied across studies, reflecting the absence of a universally accepted diagnostic standard. Among the most commonly used instruments were the Mini-Mental State Examination (MMSE), the MoCA, clinical dementia rating scales, as well as the criteria proposed by Petersen, Marilyn Albert, and Winblad. While this methodological diversity enriches the field, it also poses challenges for comparing studies and standardising diagnostic protocols.

Table 6 presents the studies that implemented various therapeutic strategies based on neurocognitive intervention modalities.

A notable diversity of approaches is evident, with conventional cognitive training being the most frequently employed modality. This intervention was delivered through both computerised formats and virtual reality, and in several cases was combined with techniques such as transcranial magnetic stimulation. These findings reflect an ongoing trend towards the use of non-invasive treatments, which are widely accepted in both clinical and experimental settings.

Most studies focused on intervening in specific cognitive functions, particularly memory (in its various forms), attention, and language. This focus suggests that these functions are the most commonly affected during the early stages of cognitive decline. Moreover, there was a consistent interest in assessing executive functions globally, highlighting the importance of considering functional performance in daily life as a key clinical criterion in the diagnosis of MCI.

Among the most relevant findings, several studies (Castellote-Caballero et al., 2024; Gozdas et al., 2024; T. M. Lee et al., 2017; Liao et al., 2020; Montero-Odasso et al., 2023; Peng et al., 2019; Wang et al., 2024) reported significant improvements in global cognitive functioning, with notable gains in attention, memory, and verbal fluency. In contrast, some trials (Carvalho et al., 2025; Jones et al., 2023) demonstrated only moderate clinical effects, while other investigations (Bray et al., 2023; Pantoni et al., 2017; Rotenberg et al., 2024; Sakurai et al., 2024) reported no significant benefits. These included interventions such as metacognitive therapy (Rotenberg et al., 2024), standalone cognitive training (Pantoni et al., 2017), a hybrid combination of physical exercise, cognitive training, and vitamin D3 supplementation (Bray et al., 2023), as well as nutritional counselling focused on cardiovascular risk management (Sakurai et al., 2024).

Table 7 presents the two studies that evaluated non-pharmacological interventions with psychotherapeutic components aimed at individuals with MCI. Both studies integrated behavioural and emotional strategies to promote cognitive and functional well-being (J. Lee et al., 2023; Rovner et al., 2018).

The first study, conducted by (Rovner et al., 2018), implemented a behavioural activation intervention in a sample of older African American adults. The intervention aimed to prevent the progression of MCI through the structured increase of meaningful activities—such as reading, walking with neighbours, and weekly phone calls—supported by personalised action plans and visual reminders. After a two-year follow-up, only 1.2% of participants in the intervention group showed memory decline, compared to 9.3% in the control group. Moreover, participants who received the behavioural activation intervention maintained their ability to carry out everyday tasks such as managing finances and using a mobile phone, whereas those in the control group experienced a decline in these functions. The study also found a significant improvement in problem-solving speed, with treated participants showing an increase of 13 s per year compared to controls.

The second study, conducted by (J. Lee et al., 2023), implemented a comprehensive programme aimed at enhancing self-efficacy by combining emotional strategies with physical and cognitive interventions. The programme included social dialogue sessions, practical workbook activities, verbal persuasion about MCI, and emotional regulation through music and physical exercise. Results indicated significant improvements in the experimental group in terms of dementia-related knowledge (F = 4.582, *p* = 0.005), levels of self-efficacy (F = 5.547, *p* = 0.002), and frequency of preventive behaviours (F = 6.376, *p* = 0.001). Additionally, the study reported an improvement in global cognitive function (F = 13.880, *p* < 0.001). However, as the intervention was multifaceted, the clinical effects could not be attributed specifically to individual components.

The eight studies presented in Table 8 employed physical interventions as a non-pharmacological therapeutic strategy. Generally, these interventions were structured as multimodal programmes incorporating aerobic, resistance, and balance exercises (Bray et al., 2023; Montero-Odasso et al., 2023). The most common frequency was two to three sessions per week, although the study by (Hassan et al., 2021) implemented a more intensive schedule of five sessions per week. Session durations ranged from 40 to 120 min. Notably, the protocol by (Buele et al., 2024) was the shortest, while that of (Steinbeisser et al., 2020) was the longest. Other studies, such as those by (Montero-Odasso et al., 2023), (Bray et al., 2023), and (J. Lee et al., 2023), maintained an average session length of 60 min.

In the case of (Montero-Odasso et al., 2023), although meaningful improvements were observed when the physical intervention was combined with other components of the experimental protocol, the effects did not reach statistical significance compared to the control group when analysed in isolation (*p* = 0.19). Similarly, the studies by (Castellote-Caballero et al., 2024), (J. Lee et al., 2023), and (Steinbeisser et al., 2020) also reported post-intervention improvements within their respective groups; however, their clinical effectiveness appears to rely more on a comprehensive approach than on physical exercise alone.

In contrast, the studies by (Buele et al., 2024) and (Hassan et al., 2021) demonstrated significant effects on cognitive variables following exclusively physical interventions. (Buele et al., 2024) reported improvements in cognitive function as measured by the MoCA, with an increase in mean scores from 20.83 (±1.8) to 23.67 (±2.24) (*p* < 0.001), alongside a reduction in depressive symptoms from 5.58 (±2.07) to 2.75 (±1.42) (*p* < 0.001). Similarly, (Hassan et al., 2021) found that resistance training led to significant cognitive gains across various neuropsychological tests, including: MoCA (from 17.60 ± 1.35 to 21.93 ± 1.57; *p* < 0.01), Mini-Mental State Examination (from 20.60 ± 1.35 to 23.20 ± 1.69; *p* < 0.01), and the Trail Making Tests A (from 1.47 ± 0.03 to 1.23 ± 0.04; *p* < 0.01) and B (from 2.51 ± 0.04 to 2.08 ± 0.04; *p* < 0.01).

Conversely, the study by (Bray et al., 2023) did not report cognitive improvements, even when physical intervention was combined with other treatments. In a different vein, (Katsipis et al., 2024) did not directly assess cognitive performance but did report improvements in biomarkers associated with the progression of MCI and inflammatory processes. Although these results do not provide direct evidence of cognitive impact, they suggest a potential neuroprotective effect of physical activity in the early stages of cognitive decline.

Table 9 presents information from recent studies that have explored the effectiveness of non-pharmacological interventions in the treatment and management of MCI. These strategies, ranging from nutritional supplementation to mind-body therapies and creative expression programmes, have been developed as alternatives or complements to conventional treatments.

In the field of nutrition, the use of vitamin D as a therapeutic supplement stands out. (Montero-Odasso et al., 2023) and (Bray et al., 2023) administered doses of 10,000 international units three times per week. However, in neither study was the effect of vitamin D evaluated in isolation as monotherapy, which limits the ability to draw definitive conclusions about its specific efficacy. In fact, Bray and colleagues did not report significant improvements in cognitive function, suggesting that, while vitamin D may play a complementary role in combined interventions, its isolated effect appears to be limited in this clinical context.

Traditional medicine has also been explored as a promising therapeutic avenue. The study by (Shin et al., 2021) examined the effects of Kami-guibi-tang, a Korean herbal formula composed of 15 medicinal plants, administered three times daily over a 24-week period. The results showed significant improvements in cognitive performance, measured by the Seoul Neuropsychological Dementia Battery, with the average score increasing from 176.00 (±24.76) at baseline to 198.47 (±31.29) at the end of the intervention (*p* < 0.001). Moreover, the treatment was well tolerated, with no reports of serious adverse effects, supporting its viability as a safe complementary therapy.

One of the most consistent findings has emerged from research on the gut-brain axis, particularly in interventions targeting the gut microbiota. In this regard, (Fei et al., 2023) assessed the daily administration of 2 g of probiotics composed of 18 different bacterial strains. The results demonstrated statistically significant improvements in cognitive function: The MMSEscore increased from 21.75 ± 2.57 to 24.75 ± 2.47 (*p* < 0.001), and the MoCA score improved from 19.80 ± 1.85 to 22.05 ± 2.14 (*p* < 0.001). The most notable improvements were observed in recall memory and visuospatial function, supporting the relevance of the gut-brain axis as a therapeutic pathway in neurodegenerative processes.

Among mind-body therapies, two approaches have shown particularly positive outcomes. The study by (Eyre et al., 2017) implemented a daily yoga routine involving mindful breathing, gentle finger movements, and moments of relaxation. This practice, carried out for just 12 min a day, resulted in significant improvements in verbal memory (d = 0.95) and in executive functions, as measured by the Trail Making Test (d = −0.75) and the Stroop Test (d = 0.71). In a separate study, (Fu & Wang, 2025) combined intensive acupuncture sessions at three cranial points—the vertex, crown, and forehead—with virtual reality training, five times per week. Although improvements were noted in MMSE and MoCA scores, the combined nature of the intervention prevents a clear attribution of effects solely to acupuncture, highlighting the need for further studies that assess its isolated impact.

Creative interventions have also emerged as effective alternatives for stimulating cognitive, emotional, and social functions. The programme designed by (Luo et al., 2024) incorporated activities such as painting, clay modelling, collage-making, and the creation of artistic narratives, delivered in one-hour sessions twice a week. This intervention led to statistically significant improvements in verbal fluency (*p* = 0.021). Complementarily, (Zhao et al., 2021) implemented a programme focused on storytelling and drawing, in which participants constructed narratives based on their illustrations and shared reflections in group settings. This had a positive impact on cognitive processing speed, a critical function in individuals with MCI.

Comparatively, interventions that address multiple dimensions of human functioning—such as probiotics, yoga, or expressive arts—tend to yield more consistent and sustained outcomes. In contrast, unidimensional approaches, such as isolated vitamin D supplementation, while safe and well-tolerated, show limitations in terms of effectiveness. Likewise, interventions that are applied with greater frequency and regularity—such as daily yoga, intensive acupuncture, or continuous probiotic intake—appear to be associated with more pronounced cognitive benefits.

As shown in Table 10, six studies were identified that evaluated the use of pharmacological interventions in individuals with MCI. Most of these studies investigated compounds with neuroprotective or antidepressant properties, aiming to exert a favourable impact on cognitive function. The pharmacological agents examined included nicotinamide riboside (Orr et al., 2024), vortioxetine (Tan & Tan, 2021), choline alfoscerate (Jeon et al., 2024), ladostigil (Schneider et al., 2019), cholinesterase inhibitors, and, in combination, donepezil with antidepressants such as citalopram and venlafaxine (Devanand et al., 2018).

Of these studies, only two reported clinically meaningful effects in terms of cognitive improvement or deceleration of decline (Jeon et al., 2024; Tan & Tan, 2021). In the first, vortioxetine was considered a promising therapeutic option; however, the authors emphasised the need to replicate the findings through randomised clinical trials, given that the absence of a comparative group limited the strength of the conclusions. The second study, focused on choline alfoscerate, demonstrated significant improvements in specific domains such as language and memory, supporting its potential use in cases of amnestic MCI.

In contrast, the clinical trial involving ladostigil (Schneider et al., 2019) showed a statistically significant reduction in global brain volume loss (*p* = 0.025) and hippocampal atrophy (*p* = 0.043); however, it failed to demonstrate clinically relevant benefits in terms of progression to dementia, based on post-treatment neuropsychological assessments. Similarly, the study involving nicotinamide riboside (Orr et al., 2024) did not report improvements in global cognitive function when compared to the placebo group.

The study that examined the combination of donepezil with citalopram and venlafaxine (Devanand et al., 2018) in participants with MCI and depressive symptoms not only showed limited clinical efficacy but also reported the highest number of adverse effects among the studies reviewed. The most frequently reported symptoms included fatigue, insomnia, headache, and dizziness, raising concerns about the safety of this combined approach in this vulnerable population.

On the other hand, the intervention that integrated cholinesterase inhibitors with a structured resistance exercise programme (Hassan et al., 2021) yielded positive results compared to the control group. However, the observed benefits were attributed primarily to the physical component of the intervention, as details regarding the specific pharmacological agent used were not reported.

Regarding the dosages administered, two studies showed variability. The nicotinamide riboside protocol (Orr et al., 2024) employed a progressive titration strategy, starting with 250 mg/day and reaching 1 g/day from the fourth week onwards. Likewise, the dosage of donepezil (Devanand et al., 2018) was adjusted according to each participant’s individual tolerance, highlighting the importance of personalised dosing strategies in the pharmacological management of MCI. In terms of safety, most studies did not report serious adverse events. The exception was the trial by Devanand et al. (Devanand et al., 2018), in which a significant burden of side effects was documented.

## 4. Discussion

Following the analysis of 40 clinical trials included in this systematic review, a sustained effort by the scientific community over the past eight years is evident in attempting to clearly define effective intervention strategies for MCI. However, the findings remain markedly heterogeneous, a fact that necessitates a more integrated and critical interpretation rather than a simple summary of interventions. This heterogeneity is not only attributable to the diversity of treatments but also to crucial methodological variations across studies.

Regarding pharmacological interventions, findings related to the use of vortioxetine and choline alphoscerate suggest a potentially beneficial effect on cognitive functions such as memory and language; nonetheless, these results remain inconclusive (Jeon et al., 2024; Tan & Tan, 2021). Conversely, compounds such as nicotinamide riboside and ladostigil did not demonstrate clinically significant benefits (Orr et al., 2024; Schneider et al., 2019), aligning with previous meta-analyses that question the efficacy of cholinesterase inhibitors in this population. Although such inhibitors may be useful in established dementia, their effectiveness in MCI appears limited and is frequently associated with adverse effects (Raschetti et al., 2007), as confirmed in the study by (Devanand et al., 2018), which reported side effects such as insomnia and dizziness with donepezil. When directly compared with non-pharmacological alternatives, current pharmacological strategies present a less favourable profile. Their limited efficacy, coupled with a higher incidence of side effects, raises significant concerns regarding long-term patient adherence and the overall risk-benefit balance. This disparity underscores the critical need for, and the growing appeal of, safer, more accessible, and patient-centred therapeutic options.

Among these alternatives, cognitive training—delivered through conventional methods, computer-based platforms, or virtual reality—has emerged as the most effective non-pharmacological intervention in slowing cognitive decline. Several studies have reported significant improvements in domains such as memory, attention, executive function, and global cognition (Gozdas et al., 2024; Liao et al., 2020). These results are consistent with previous meta-analyses supporting the efficacy of such interventions (Sung et al., 2023a), and highlight their non-invasive nature as a key factor in patient acceptance and adherence, due to their perceived low risk, accessibility, and safety. However, studies such as that by (Sakurai et al., 2024) reported no significant benefits following a multidomain intervention, possibly due to factors such as treatment intensity, duration, or participant adherence, which emphasises the importance of optimising not only the intervention strategies themselves but also the conditions under which they are implemented.

Furthermore, the mixed or inconclusive evidence for some interventions requires a nuanced discussion. The failure of a multidomain intervention to show benefits, as in the study by (Sakurai et al., 2024), should not lead to a blanket dismissal of the approach. Instead, it compels an analysis of plausible explanatory factors, such as insufficient treatment intensity, short duration, or low participant adherence—variables that are critical to an intervention’s success. Similarly, while psychotherapy is not a primary treatment for the cognitive symptoms of MCI, its potential is highlighted by the high comorbidity with depression. The innovative behavioural activation protocol used by (Rovner et al., 2018), which differed from standard depression treatments, succeeded in maintaining daily living skills.

From a clinical and public health perspective, these findings have clear implications. There is a strong rationale for prioritising non-pharmacological interventions that are low-risk, highly accessible, and can be integrated into a patient’s lifestyle. Cognitive training, structured physical exercise, and dietary modifications represent feasible first-line strategies in both preventive and clinical contexts. It is vital to recognise that even modest improvements in cognitive performance can have a profound impact on an individual’s daily life. Maintaining the ability to manage finances, use a telephone, or live independently, as seen in the study (Rovner et al., 2018), represents a clinically meaningful outcome that preserves quality of life and functional autonomy.

A key insight emerging from this review is that the most promising interventions are not isolated but rather synergistic and multimodal. The evidence suggests that combining approaches may yield superior outcomes. For instance, two studies demonstrated that transcranial magnetic stimulation (TMS) was effective when paired with cognitive and physical tasks, improving multiple cognitive domains (Malavera et al., 2014; Yan et al., 2023). This aligns with a broader principle seen in physical therapy, which also showed greater effects on global cognition and neurodegeneration biomarkers when combined with cognitive training (Buele et al., 2024; Katsipis et al., 2024). These findings are consistent with the results of (Longhurst et al., 2020), who observed improvements in attention and memory following one month of physiotherapy. Likewise, the meta-analysis by (H. Li et al., 2022) highlighted the benefits of resistance training in individuals over 60 years of age, particularly in enhancing attention and executive functions. Nonetheless, reviews such as that by (Kaufman et al., 2024) suggest that the effects of short or moderate-intensity physical interventions may not be sustainable over time; thus, promoting an active lifestyle appears to be a more effective strategy for maintaining cognitive function in this population. These strongly suggest that successful interventions often share common features: they are active rather than passive, often multimodal, and their impact is likely modulated by factors such as treatment intensity, duration, and personalisation to the patient’s specific deficits. Targeting specific brain regions, like the dorsolateral prefrontal cortex, with TMS, further exemplifies this move towards more tailored and precise therapeutic strategies.

Other, less explored interventions, such as psychotherapy, have also shown potential in this area. Although not considered a first-line treatment for MCI, studies have documented high comorbidity with emotional disorders such as depression (Pellegrino et al., 2013), supporting the inclusion of psychotherapy within a comprehensive therapeutic approach. In this context, the study by (Rovner et al., 2018) stands out: using a behavioural activation protocol, the authors found a significant maintenance of cognitive functions and daily living skills—such as mobile phone use and financial management—among African American patients with MCI, compared to the control group. It is important to note that this protocol differs from the standard model commonly used in treating depressive disorders (Cuijpers et al., 2023), making it an innovative proposal worthy of further research attention.

Similarly, complementary therapies such as the use of probiotics, yoga practice, and the consumption of Korean herbal beverages have yielded promising preliminary results in slowing MCI progression (Eyre et al., 2017; Fei et al., 2023; Shin et al., 2021), in some cases surpassing those of pharmacological treatments. From this perspective, such findings call for a reconsideration of current paradigms concerning the aetiology, diagnosis, and treatment of MCI. For example, the benefits of probiotics observed in this review are consistent with the meta-analysis by (Xiao et al., 2025), which identified cognitive improvements in individuals with both Alzheimer’s disease and MCI. Likewise, the cognitive benefits of yoga reported here are supported by the systematic review by (Karamacoska et al., 2023), while improvements associated with Korean herbal beverages are in line with the findings of (Kumar et al., 2013), who reported enhanced cognitive performance linked to several Southeast Asian herbal formulas in individuals with MCI.

## 5. Limitations

When interpreting the findings of this review, several methodological limitations should be taken into account. The considerable heterogeneity across the included studies—in terms of design, duration, diagnostic criteria, assessment tools, and sample sizes—limited the feasibility of conducting a robust meta-analysis and complicated systematic comparisons.

In addition, a number of trials exhibited a high risk of bias in key areas such as randomisation, blinding, and outcome assessment, which weakens the overall reliability of the conclusions. Many of the studies were exploratory or at an early stage of development, preventing the establishment of clear causal relationships and requiring the findings to be viewed as tentative associations.

Another relevant concern is the limited availability of longitudinal data, which restricts the ability to assess the long-term impact of the interventions. Therefore, caution is advised when generalising these results, and there is a clear need for more rigorous research with stronger methodological controls, extended follow-up periods, and more representative samples to enhance future clinical recommendations in the field of MCI.

## 6. Conclusions

This systematic review highlights the need to move beyond conventional approaches to the treatment of MCI, emphasising the value of non-pharmacological interventions as a primary means of preserving cognitive function. Strategies such as cognitive training, regular physical activity, and behavioural stimulation have shown tangible benefits, both in slowing cognitive decline and in restoring key cognitive abilities, with fewer associated risks than traditional pharmacological treatments.

Complementary practices such as yoga, the use of probiotics, and the incorporation of herbal infusions also offer promising preliminary evidence. Although further scientific validation is required, these approaches point towards a broader and more integrative model of care, extending beyond a strictly biomedical perspective.

The current direction in MCI management thus appears to favour more person-centred strategies, where tailored interventions, long-term sustainability, and the enhancement of quality of life take precedence. This implies acknowledging the human dimension of cognitive processes by integrating emotional, social, and behavioural aspects that directly shape the lived experience of individuals affected by MCI.

## Figures and Tables

**Table 1:** PICO Component Description.

Population (P)	Adults aged 50 years or older with MCI.
Intervention (I)	Pharmacological: Medications such as cholinesterase inhibitors, memantine, etc.
	Non-pharmacological: Cognitive therapies, physical exercise, dietary interventions, cognitive stimulation.
Comparison (C)	Placebo, standard care, or no intervention.
Outcomes (O)	Primary: Improvement in cognition (assessed through standardised cognitive tests).
	Secondary: Improvement in function (activities of daily living), behaviour (neuropsychiatric symptoms), general status (quality of life), and mortality.

**Table 2:** Descriptors used in the search strategy.

Term	DeCS/MeSH
Pharmacological intervention	Drug Therapy, Pharmacologic Treatment, Medication Therapy
Non-pharmacological intervention	Non-Pharmacologic Therapy, Non-Drug Treatment, Behavioural Therapy
Mild cognitive impairment	Cognitive Dysfunction, Mild Cognitive Impairment, Cognitive Decline
Older adults	Aged, Elderly, Older Adults
Cognition	Cognition, Knowledge, Understanding
Behaviour	Behaviour, Conduct, Attitude
Mortality	Mortality Rate, Fatality, Death Rate

**Table 3:** Search algorithms used.

No.		Algorithm
1	PubMed	(“Drug Therapy” OR “Pharmacologic Treatment” OR “Medication Therapy” OR “NMDA Receptor Antagonists” OR “Memantine” OR “Cognitive Rehabilitation” OR “Cognitive Remediation” OR “Cognitive Training” OR “Behavioural Therapy”) AND (“Cognition” OR “Knowledge” OR “Understanding” OR “Behaviour” OR “Conduct” OR “Attitude” OR “Mortality Rate” OR “Fatality” OR “Death Rate” OR “Cognitive Dysfunction” OR “Mild Cognitive Impairment” OR “Cognitive Decline”) AND (“Aged” OR “Elderly” OR “Older Adults”)
2	Scopus	(“Drug Therapy” OR “Pharmacologic Treatment” OR “Medication Therapy” OR “NMDA Receptor Antagonists” OR “Memantine”) AND (“Cognition” OR “Knowledge” OR “Understanding” OR “Behaviour” OR “Conduct” OR “Attitude” OR “Mortality Rate” OR “Fatality” OR “Death Rate” OR “Cognitive Dysfunction” OR “Mild Cognitive Impairment” OR “Cognitive Decline”) AND (“Aged” OR “Elderly” OR “Older Adults”)
3	ScienceDirect	(“Behavioural Therapy” OR “Cognitive Therapy” OR “Cognitive Behavioural Therapy” OR “Cognitive Rehabilitation” OR “Cognitive Remediation”) AND (“Cognitive Dysfunction” OR “Mild Cognitive Impairment” OR “Cognitive Decline” OR “Cognition” OR “Knowledge” OR “Understanding” OR “Behaviour” OR “Conduct” OR “Attitude” OR “Mortality Rate” OR “Fatality” OR “Death Rate”) AND (“Aged” OR “Elderly” OR “Older Adults”)
4	SpringerLink	(“Cognitive Rehabilitation” OR “Cognitive Remediation” OR “Cognitive Training” OR “Behavioural Therapy”) AND (“Cognitive Dysfunction” OR “Mild Cognitive Impairment” OR “Cognitive Decline”) AND (“Older Adults” OR “Elderly”)

Note: The search algorithms were tailored and adapted to the specific syntax and indexing terms of the databases PubMed, Scopus, ScienceDirect, SpringerLink, and Web of Science (WOS).

**Table 4:** Compilation of Selected Studies.

Database	Total Found	Type of Document	Time Period	No Access	Reviews/Incomplete Texts/Duplicates	Non-Compliance with Variable Criteria	Final Sample
PubMed	75,900	58,932	10,899	2553	0	3495	20
Scopus	22,731	2382	11,756	3682	2	4910	0
ScienceDirect	5330	3262	670	718	1	666	13
SpringerLink	3845	1950	943	9	0	940	3
WOS	894	242	162	166	0	320	4
Total	108,700	66,768	24,430	7128	3	10,331	40

Note: Database: source of search; Total found: total records retrieved; Document type: records by publication type filter; Time period: records within the study’s inclusion date range (count, not duration); No access: full text unavailable; Reviews/Incomplete/Duplicates: excluded for being reviews, incomplete texts, or duplicates; Non-compliance: excluded for not meeting variable criteria; Final sample: studies included after all exclusions.

**Table 5:** Complete Summary of Included Studies (n = 40).

Author(s) (Year)	Country	Design	Duration	Intervention	Sample (n)	Key Findings	Clinically Significant?	**Diagnostic Criteria**
(Liao et al., 2020)	Taiwan	RCT	12 weeks	VR training	34	Executive function gains	Yes, *p* < 0.001, in global cognition	MMSE, MoCA
(Montero-Odasso et al., 2023)	Canada	RCT	20 weeks	Multimodal	175	Memory gains	Yes, *p* < 0.005, Cohen’s d = 0.71 in global cognition	MoCA, Albert
(Sakurai et al., 2024)	Japan	RCT	78 weeks	Multimodal	433	No prevention	No, *p* = 0.226, Cohen’s d = 0.087, in global cognition	MMSE, Weschler
(Peng et al., 2019)	China	RCT	26 weeks	Cognitive training	140	Cognitive gains	Yes, *p* < 0.00,1, ηp^2^ = 0.295, in global cognition	MoCA
(Orr et al., 2024)	USA	RCT	10 weeks	Nicotinamide	20	No change	No, *p* = 0.57, in global cognition	MoCA
(Gozdas et al., 2024)	USA	RCT	26 weeks	Multidomain	34	Brain connectivity	Yes, *p* = 0.007, in global cognition	MMSE, Weschler
(T. M. Lee et al., 2017)	Hong Kong	RCT	13 weeks	Auditory training	239	Improved attention/memory	Yes, *p* < 0.001, in global cognition	MoCA
(Tan & Tan, 2021)	Singapore	Single-arm	26 weeks	Vortioxetine	111	Cognitive gains	Yes, *p* < 0.001, in global cognition	MoCA, CDR
(Wang et al., 2024)	China	RCT	7 weeks	Lifestyle program	123	Significant gains	Yes, *p* = 0.002, Cohen’s d = 0.63, in global cognition	MMSE, MoCA
(Jeon et al., 2024)	South Korea	RCT	12 weeks	Choline supplement	100	Cognitive gains	Yes, *p* = 0.048, in global cognition	ADAS-cog, MoCA
(Castellote-Caballero et al., 2024)	Spain	RCT	12 weeks	Physical + cognitive	95	Global cognition	Yes, *p* = 0.041, in global cognition	MMSE
(Shin et al., 2021)	South Korea	RCT	24 weeks	Herbal treatment	30	Beat placebo	Yes, *p* = 0.045, in global cognition	Seoul Battery
(Lau et al., 2024)	Taiwan	RCT	5 weeks	tDCS + training	21	Better gait	Yes, *p* < 0.001, in global cognition	Petersen
(Rovner et al., 2018)	USA	RCT	16 weeks	Behavioural therapy	221	Improved attention	Yes, *p* = 0.002, (RR) 0.12 (IC 95%: 0.02–0.74) decline in memory	Hopkins tests
(Buele et al., 2024)	Ecuador	RCT	6 weeks	VR + physical	26	Less depression	Yes, *p* < 0.001, Cohen’s = 1.54, in global cognition	MoCA
(Schneider et al., 2019)	Multinational	RCT	156 weeks	Ladostigil	202	No delay in dementia	No, *p* = 0.426, in global cognition	MMSE, CDR
(B.-Y. Y. Li et al., 2019)	China	RCT	26 weeks	Computerised training	141	Short-term gains	Moderate, *p* = 0.002, in global cognition (this effect was not sustained in the long term)	MMSE, ATN
(Baik et al., 2024)	South Korea	RCT	8 weeks	Computerised	50	Less depression	Yes, (F = 34.44, *p* < 0.001), in global cognition and group differences	MoCA, MMSE
(Sung et al., 2023b)	Taiwan	RCT	8 weeks	Multidomain	72	Coordination	Yes, (β = 1.47; IC 95% = 0.63–2.31; *p* = 0.001), in global cognition	CDR
(Bray et al., 2023)	Canada	RCT	20 weeks	Exercise + D3	90	Minimal effects	No, *p* > 0.05, in global cognition.	Albert criteria
(Pantoni et al., 2017)	Italy	RCT	20 weeks	Cognitive training	43	No improvements	No, *p* = 0.381, in global cognition	Winblad criteria
(Rotenberg et al., 2024)	Canada	RCT	10 weeks	Metacognitive	264	No benefits	No, *p* = 0.775, Cohen’s d = −0.06 to 0.15, in global cognition	Hopkins tests
(Luo et al., 2024)	China	RCT	12 weeks	Arts program	38	Verbal fluency	Yes, *p* = 0.021, in verbal fluency	Petersen, MoCA
(Park, 2022)	South Korea	RCT	8 weeks	VR spatial training	56	Spatial memory	Yes, *p* < 0.001 in memory spatial, *p* < 0.05 in episodic memory	MMSE, CVLT
(Motter et al., 2024)	USA	RCT	12 weeks	Computerised training	107	Global improvements	Yes, *p* < 0.05, in global cognition	MMSE
(J. Lee et al., 2023)	South Korea	RCT	8 weeks	Self-efficacy	32	Dementia knowledge	Yes, (F = 13.880, *p* < 0.001), in global cognition	Petersen
(Fei et al., 2023)	China	RCT	12 weeks	Probiotics	42	Sleep/cognition	Yes, *p* < 0.001, in global cognition	Petersen, MMSE
(Katsipis et al., 2024)	Greece	RCT	12 weeks	Exercise + training	53	Biomarker changes	Yes, *p* < 0.05 en IL-1β, IL-6, *p*-tau181/Aβ42	Petersen, MMSE
(Wu et al., 2023)	China	RCT	8 weeks	Computerised	53	Episodic memory	Yes, *p* = 0.008 in immediate memory, *p* = 0.009 in delayed recall	Petersen, CDR
(Nwosu et al., 2024)	USA	RCT	78 weeks	Computerised	105	Racial differences	Yes, *p* = 0.009 compared to whites	Not specified
(Nakagawa et al., 2024)	Japan	Single-arm	34 weeks	Multimodal	68	Maintained function	Yes, *p* = 0.020 in CFI, no improvement in the MMSE *p* = 0.147	MMSE
(Fu & Wang, 2025)	China	RCT	8 weeks	VR + acupuncture	46	Memory	Yes, Z = 3.38 (*p* < 0.0001), in global cognition	Petersen, MMSE
(Eyre et al., 2017)	USA	RCT	12 weeks	Yoga	81	Better executive function	Yes, F (2,74) = 3.24, *p* = 0.04, in executive function	MMSE, Hopkins
(Carvalho et al., 2025)	Brazil	RCT	Unspecified	Digital	66	Selective gains	Moderate, *p* = 0.01, F (1,45) = 7.07, d = 0.79 in functional performance, but with no effect on overall cognition	MoCA, Stroop
(Jones et al., 2023)	USA	RCT	4 weeks	tDCS + training	27	Mixed attention	Moderate, F (1.31, 23.56) = 6.25, *p* = 0.014 in attention, but no effect on memory	MoCA, CVLT
(Prokopenko et al., 2019)	Russia	RCT	Unspecified	Computerised training	68	Better processing speed	Yes, *p* = 0.006 in global cognition	MMSE, FAB
(Hassan et al., 2021)	Pakistan	Quasi-exp.	6 weeks	Exercise + meds	30	Memory gains	Yes, *p* = 0.00, in global cognition	MMSE, MoCA
(Zhao et al., 2021)	China	RCT	16 weeks	Creative arts	36	Attention gains	Yes, *p* = 0.011, in reaction times	DSM-IV, MoCA
(Devanand et al., 2018)	USA	RCT	62 weeks	Donepezil combo	61	No benefits	No, *p* = 0.13, in global cognition	Weschler Memory
(Steinbeisser et al., 2020)	Germany	RCT	26 weeks	Multimodal	433	Daily function	Yes, *p* = 0.02 (95% CI): 0.17 to 1.67, in global cognition	MMSE, ADL

**Table 6:** Cognitive Interventions and Assessed Domains (n = 29).

Author(s) (Year)	Intervention Type	Cognitive Domains Assessed
(T. M. Lee et al., 2017)	Auditory cognitive training	Sustained auditory attention, visual attention, visuospatial working memory
(Pantoni et al., 2017)	Cognitive training	Verbal fluency, memory, executive functions, attention
(Sakurai et al., 2024)	Cognitive training	Memory, attention, executive functions, processing speed
(Peng et al., 2019)	Cognitive training	Attention, language, abstraction, delayed recall, orientation, naming
(B.-Y. Y. Li et al., 2019)	Computerised cognitive training	Attention, memory, language, visuospatial skills, processing speed, executive functions
(Prokopenko et al., 2019)	Computerised cognitive training	Attention, visual memory, visuospatial abilities
(Motter et al., 2024)	Computerised cognitive training	Episodic memory, working memory, daily functioning, global cognition
(Liao et al., 2020)	Cognitive training	Global cognition, executive functions, verbal memory
(Park, 2022)	Virtual reality cognitive training	Spatial cognition, episodic memory
(Steinbeisser et al., 2020)	Cognitive training	Global cognitive function
(Katsipis et al., 2024)	Cognitive training	Attention, language, executive functions
(Castellote-Caballero et al., 2024)	Cognitive training	Verbal fluency, executive functions, memory, attention, language, orientation, abstraction, visuospatial skills
(Gozdas et al., 2024)	Cognitive training	Executive functions, inhibitory control, episodic memory, working memory, processing speed
(Wang et al., 2024)	Cognitive training	Memory, attention, executive functions
(Buele et al., 2024)	Virtual reality cognitive training	Attention, verbal fluency, executive functions, short-term memory, visuospatial memory, language, temporal/spatial orientation, calculation
(Lau et al., 2024)	Computerised training + tDCS	Working memory, episodic memory (Stimulation: Left frontal cortex, 2 mA, 20 min/session)
(Rotenberg et al., 2024)	Metacognitive therapy	Verbal memory, visuospatial memory, executive functions
(Nakagawa et al., 2024)	Cognitive stimulation	Attention, memory, visuospatial function, language, reasoning
(Nwosu et al., 2024)	Computerised cognitive training	Memory, functional abilities
(Baik et al., 2024)	Computerised cognitive training	Sustained attention, selective attention, visual perception, memory, executive functions
(Sung et al., 2023b)	Multidomain cognitive training	Working memory, selective attention, visuospatial attention, divided attention, coordination
(Bray et al., 2023)	Cognitive training	Working memory, attention, executive functions
(Montero-Odasso et al., 2023)	Computerised cognitive training	Memory, orientation, attention, language, executive functions, praxis
(J. Lee et al., 2023)	Cognitive training	Orientation, abstraction, language, attention, naming, visuospatial memory, delayed recall
(Wu et al., 2023)	Computerised cognitive training	Episodic memory, attention, working memory, response speed
(Jones et al., 2023)	Cognitive training + tDCS	Working memory, attentional orientation, visuospatial search, inhibitory control (Stimulation: Prefrontal cortex, 1.5 mA, 15 min/session)
(Fu & Wang, 2025)	Virtual reality training	Verbal tracking, abstract thinking, naming ability, visual/executive function
(Carvalho et al., 2025)	Digital cognitive training	Executive functions, memory, language, visuospatial skills, attention, orientation, processing speed, inhibitory control

**Table 7:** Non-Pharmacological Interventions (Psychotherapeutic Approaches).

Author(s) (Year)	Intervention Type	Behavioural Strategy	Sample (n)	Key Findings	Clinically Significant?
(Rovner et al., 2018)	Behavioural activation	- Personalised action plan (reading, walking with neighbours, weekly calls) - Visual reminders for activity execution	221	Improved attention/mental flexibility	Yes
(J. Lee et al., 2023)	Self-efficacy enhancement	- Verbal persuasion about MCI - Social dialogue spaces - Practical workbook activities - Emotional regulation through music and physical exercises	32	Increased dementia knowledge/confidence	Yes

**Table 8:** Non-Pharmacological Physical Interventions (Complete).

Author(s) (Year)	Country	Intervention Type	Physical Activity Description	Frequency
(Steinbeisser et al., 2020)	Germany	Motor stimulation	Balance exercises, balloon games, gross motor skill activities	2-h daily sessions
(Hassan et al., 2021)	Pakistan	Resistance training	Dumbbell and elastic band exercises	5×/week, 50 min/session
(Montero-Odasso et al., 2023)	Canada	Aerobic + resistance	Treadmill walking, static cycling, weight lifting, elastic bands	3×/week, 60 min/session
(Bray et al., 2023)	Canada	Physical exercise	Running, static cycling, leg press, hamstring curls, chest press, balance/stretching	3×/week, 60 min/session
(J. Lee et al., 2023)	South Korea	Physical activity	Not specified	1×/week, 60 min/session
(Castellote-Caballero et al., 2024)	Spain	Physical exercise	Slow walking, chair exercises (heel lifts, torso twists), ball passes, agility circuits, balance/stretching	2×/week, 45–50 min/session
(Buele et al., 2024)	Ecuador	Physical exercise	Balance exercises, squats, brisk walking, stair climbing	2×/week, 40 min/session
(Katsipis et al., 2024)	Greece	Physical exercise	Head/neck movements, shoulder exercises, dual hand-foot tasks	2–3×/week, 45 min/session

**Table 9:** Non-Pharmacological (Alternative) Interventions.

Author(s) (Year)	Country	Intervention Type	Procedure Details	Duration	Frequency
(Eyre et al., 2017)	USA	Kundalini yoga	Breathing techniques, finger movements, and rest periods	12 weeks	12-min sessions daily
(Zhao et al., 2021)	China	Creative expression (narrative-based)	Interactive games, drawing, story creation from artwork, group discussions	16 weeks	Not specified
(Shin et al., 2021)	South Korea	Kami-guibi-tang (herbal formula)	3 g herbal granules (15 medicinal herbs) dissolved in warm water, 3× daily after meals	24 weeks	3 doses/day
(Fei et al., 2023)	China	Probiotics	2 g daily probiotic blend (18 bacterial strains)	12 weeks	Daily
(Montero-Odasso et al., 2023)	Canada	Vitamin D supplementation	10,000 IU three times weekly	20 weeks	3×/week
(Bray et al., 2023)	Canada	Vitamin D3 supplementation	10,000 IU three times weekly	20 weeks	3×/week
(Luo et al., 2024)	China	Intensive creative arts programme	Visual arts (painting, collage) + narrative storytelling	12 weeks	60-min sessions 2×/week
(Fu & Wang, 2025)	China	Acupuncture	Fine needles at three head points (vertex, crown, forehead)	8 weeks	30-min sessions 5×/week

**Table 10:** Pharmacological Intervention Studies.

Author(s) (Year)	Intervention	Dosage	Safety Profile
(Devanand et al., 2018)	Donepezil + Citalopram/Venlafaxine	Donepezil: 5 mg/day (titrated to 10 mg/day)	Moderate adverse events: diarrhoea, headaches, fatigue, insomnia, nightmares, dizziness
(Schneider et al., 2019)	Ladostigil	10 mg/day	No serious drug-related adverse events reported
(Tan & Tan, 2021)	Vortioxetine	5 mg/day	No serious drug-related adverse events reported
(Hassan et al., 2021)	Cholinesterase inhibitors	Not specified	Not specified
(Orr et al., 2024)	Nicotinamide riboside	Week 1: 250 mg/day; Week 2: 500 mg/day; Week 3: 750 mg/day; Week 4+: 1 g/day	No serious drug-related adverse events reported
(Jeon et al., 2024)	Choline alphoscerate	600 mg/day	No serious drug-related adverse events reported

## Data Availability

No new data were created or analyzed in this study. Data sharing is not applicable to this article.

## References

[B1-ejihpe-15-00226] Andrango Pilataxi M. L., López Barba D. F. (2022). Abordaje clínico del deterioro cognitivo leve en atención primaria. RECIMUNDO: Revista Científica de la Investigación y el Conocimiento.

[B2-ejihpe-15-00226] Apostolova L. G., Di L. J., Duffy E. L., Brook J., Elashoff D., Tseng C. H., Fairbanks L., Cummings J. L. (2014). Risk factors for behavioral abnormalities in mild cognitive impairment and mild Alzheimer’s disease. Dementia and Geriatric Cognitive Disorders.

[B3-ejihpe-15-00226] Baik J. S., Min J. H., Ko S.-H., Yun M. S., Lee B., Kang N. Y., Kim B., Lee H., Shin Y.-I. (2024). Effects of home-based computerized cognitive training in community-dwelling adults with mild cognitive impairment. IEEE Journal of Translational Engineering in Health and Medicine.

[B4-ejihpe-15-00226] Bidzan L., Grabowski J., Przybylak M., Ali S. (2023). Aggressive behavior and prognosis in patients with mild cognitive impairment. Dementia & Neuropsychologia.

[B5-ejihpe-15-00226] Bray N. W., Pieruccini-Faria F., Witt S. T., Bartha R., Doherty T. J., Nagamatsu L. S., Almeida Q. J., Liu-Ambrose T., Middleton L. E., Bherer L., Montero-Odasso M. (2023). Combining exercise with cognitive training and vitamin D3 to improve functional brain connectivity (FBC) in older adults with mild cognitive impairment (MCI). Results from the SYNERGIC trial. GeroScience.

[B6-ejihpe-15-00226] Buckinx F., Aubertin-Leheudre M. (2021). Nutrition to prevent or treat cognitive impairment in older adults: A GRADE recommendation. The Journal of Prevention of Alzheimer’s Disease.

[B7-ejihpe-15-00226] Buele J., Avilés-Castillo F., Del-Valle-Soto C., Varela-Aldás J., Palacios-Navarro G. (2024). Effects of a dual intervention (motor and virtual reality-based cognitive) on cognition in patients with mild cognitive impairment: A single-blind, randomized controlled trial. Journal of NeuroEngineering and Rehabilitation.

[B8-ejihpe-15-00226] Carcelén-Fraile M. D. C., Llera-DelaTorre A. M., Aibar-Almazán A., Afanador-Restrepo D. F., Baena-Marín M., Hita-Contreras F., Brandão-Loureiro V., García-Garro P. A., Castellote-Caballero Y. (2022). Cognitive stimulation as alternative treatment to improve psychological disorders in patients with mild cognitive impairment. Journal of Clinical Medicine.

[B9-ejihpe-15-00226] Carvalho C. M., Poltronieri B. C., Reuwsaat K., Reis M. E. A., Panizzutti R. (2025). Digital cognitive training for functionality in mild cognitive impairment: A randomized controlled clinical trial. GeroScience.

[B10-ejihpe-15-00226] Castellote-Caballero Y., Carcelén Fraile M. D. C., Aibar-Almazán A., Afanador-Restrepo D. F., González-Martín A. M. (2024). Effect of combined physical-cognitive training on the functional and cognitive capacity of older people with mild cognitive impairment: A randomized controlled trial. BMC Medicine.

[B11-ejihpe-15-00226] Chertkow H., Massoud F., Nasreddine Z., Belleville S., Joanette Y., Bocti C., Drolet V., Kirk J., Freedman M., Bergman H. (2008). Diagnosis and treatment of dementia: 3. Mild cognitive impairment and cognitive impairment without dementia. Canadian Medical Association Journal.

[B12-ejihpe-15-00226] Cuijpers P., Karyotaki E., Harrer M., Stikkelbroek Y. (2023). Individual behavioral activation in the treatment of depression: A meta analysis. Psychotherapy Research.

[B13-ejihpe-15-00226] Devanand D. P., Pelton G. H., D’Antonio K., Ciarleglio A., Scodes J., Andrews H., Lunsford J., Beyer J. L., Petrella J. R., Sneed J., Ciovacco M., Doraiswamy P. M. (2018). Donepezil treatment in patients with depression and cognitive impairment on stable antidepressant treatment: A randomized controlled trial. The American Journal of Geriatric Psychiatry.

[B14-ejihpe-15-00226] Devier D. J., Villemarette-Pittman N., Brown P., Pelton G., Stern Y., Sano M., Devanand D. P. (2010). Predictive utility of type and duration of symptoms at initial presentation in patients with mild cognitive impairment. Dementia and Geriatric Cognitive Disorders.

[B15-ejihpe-15-00226] Eyre H. A., Siddarth P., Acevedo B., Van Dyk K., Paholpak P., Ercoli L., St Cyr N., Yang H., Khalsa D. S., Lavretsky H. (2017). A randomized controlled trial of Kundalini yoga in mild cognitive impairment. International Psychogeriatrics.

[B16-ejihpe-15-00226] Fei Y., Wang R., Lu J., Peng S., Yang S., Wang Y., Zheng K., Li R., Lin L., Li M. (2023). Probiotic intervention benefits multiple neural behaviors in older adults with mild cognitive impairment. Geriatric Nursing.

[B17-ejihpe-15-00226] Feldman H. H., Jacova C. (2005). Mild cognitive impairment. The American Journal of Geriatric Psychiatry: Official Journal of the American Association for Geriatric Psychiatry.

[B18-ejihpe-15-00226] Fitzpatrick-Lewis D., Warren R., Ali M. U., Sherifali D., Raina P. (2015). Treatment for mild cognitive impairment: A systematic review and meta-analysis. CMAJ Open.

[B19-ejihpe-15-00226] Fu Y., Wang H. (2025). Clinical observation of VR virtual reality rehabilitation training combined with acupuncture in the treatment of mild cognitive impairment. SLAS Technology.

[B20-ejihpe-15-00226] Ginsberg T. B., Powell L., Emrani S., Wasserman V., Higgins S., Chopra A., Cavalieri T. A., Libon D. J. (2019). Instrumental activities of daily living, neuropsychiatric symptoms, and neuropsychological impairment in mild cognitive impairment. Journal of the American Osteopathic Association.

[B21-ejihpe-15-00226] González Hernández A., Rodríguez Quintero A. M., Bonilla Santos J. (2022). Depression and its relationship with mild cognitive impairment and Alzheimer disease: A review study. Revista Espanola de Geriatria y Gerontologia.

[B22-ejihpe-15-00226] González Martínez P., Oltra Cucarella J., Sitges Maciá E., Bonete López B. (2021). Revisión y actualización de los criterios de deterioro cognitivo objetivo y su implicación en el deterioro cognitivo leve y la demencia. Revista de Neurología.

[B23-ejihpe-15-00226] Gozdas E., Avelar-Pereira B., Fingerhut H., Dacorro L., Jo B., Williams L., O’Hara R., Hosseini S. M. H. (2024). Long-term cognitive training enhances fluid cognition and brain connectivity in individuals with MCI. Translational Psychiatry.

[B24-ejihpe-15-00226] Guerrero Barragán A., Lucumí Cuesta D. I., Gómez I. E., Lawior B. (2023). Análisis situacional del deterioro cognitivo en Colombia. Notas de Política.

[B25-ejihpe-15-00226] Hale J. M., Schneider D. C., Mehta N. K., Myrskylä M. (2020). Cognitive impairment in the U.S.: Lifetime risk, age at onset, and years impaired. SSM—Population Health.

[B26-ejihpe-15-00226] Hassan M., Rashid S., Khan R. R., Khalid M. U., Mansha H., Khalid H. (2021). Effects of structured resisted exercises on cognition level among patients with mild cognitive impairment. Pakistan Journal of Medical and Health Sciences.

[B27-ejihpe-15-00226] Hu C., Yu D., Sun X., Zhang M., Wang L., Qin H. (2017). The prevalence and progression of mild cognitive impairment among clinic and community populations: A systematic review and meta-analysis. International Psychogeriatrics.

[B28-ejihpe-15-00226] Hughes T. F., Snitz B. E., Ganguli M. (2011). Should mild cognitive impairment be subtyped?. Current Opinion in Psychiatry.

[B29-ejihpe-15-00226] Jak A. J., Bondi M. W., Delano-Wood L., Wierenga C., Corey-Bloom J., Salmon D. P., Delis D. C. (2009). Quantification of five neuropsychological approaches to defining mild cognitive impairment. The American Journal of Geriatric Psychiatry.

[B30-ejihpe-15-00226] Jefferson A. L., Beiser A. S., Seshadri S., Wolf P. A., Au R. (2015). APOE and mild cognitive impairment: The Framingham heart study. Age and Ageing.

[B31-ejihpe-15-00226] Jeon J., Lee S. Y., Lee S., Han C., Park G. D., Kim S. J., Chang J. G., Kim W. J. (2024). Efficacy and safety of choline alphoscerate for amnestic mild cognitive impairment: A randomized double-blind placebo-controlled trial. BMC Geriatrics.

[B32-ejihpe-15-00226] John A., Patel U., Rusted J., Richards M., Gaysina D. (2019). Affective problems and decline in cognitive state in older adults: A systematic review and meta-analysis. Psychological Medicine.

[B33-ejihpe-15-00226] Jones K. T., Ostrand A. E., Gazzaley A., Zanto T. P. (2023). Enhancing cognitive control in amnestic mild cognitive impairment via at-home non-invasive neuromodulation in a randomized trial. Scientific Reports.

[B34-ejihpe-15-00226] Karamacoska D., Tan T., Mathersul D. C., Sabag A., de Manincor M., Chang D., Steiner-Lim G. Z. (2023). A systematic review of the health effects of yoga for people with mild cognitive impairment and dementia. BMC Geriatrics.

[B35-ejihpe-15-00226] Katsipis G., Tzekaki E. E., Andreadou E. G., Mouzakidis C., Baldimtsi E. N., Karathanasi E. M., Hassandra M., Galanis E., Hatzigeorgiadis A., Goudas M., Zikas P., Evangelou G., Papagiannakis G., Bellis G., Kokkotis C., Tsatalas T., Giakas G., Theodorakis Y., Tsolaki M., Pantazaki A. A. (2024). The effect of physical exercise with cognitive training on inflammation and Alzheimer’s disease biomarkers of Mild Cognitive Impairment patients. Neuroscience Applied.

[B36-ejihpe-15-00226] Katsuno M., Sahashi K., Iguchi Y., Hashizume A. (2018). Preclinical progression of neurodegenerative diseases. Nagoya Journal of Medical Science.

[B37-ejihpe-15-00226] Kaufman M., Dyrek P., Fredericson M., Oppezzo M., Roche M., Frehlich L., Noordsy D. (2024). The role of physical exercise in cognitive preservation: A systematic review. American Journal of Lifestyle Medicine.

[B38-ejihpe-15-00226] Knopman D. S., Petersen R. C. (2014). Mild cognitive impairment and mild dementia: A clinical perspective. Mayo Clinic Proceedings.

[B39-ejihpe-15-00226] Kumar H., Song S.-Y., More S., Kang S.-M., Kim B.-W., Kim I.-S., Choi D.-K. (2013). Traditional Korean East Asian medicines and herbal formulations for cognitive impairment. Molecules.

[B40-ejihpe-15-00226] Lau C. I., Liu M.-N., Cheng F.-Y., Wang H.-C., Walsh V., Liao Y.-Y. (2024). Can transcranial direct current stimulation combined with interactive computerized cognitive training boost cognition and gait performance in older adults with mild cognitive impairment? a randomized controlled trial. Journal of NeuroEngineering and Rehabilitation.

[B41-ejihpe-15-00226] Lee J., Cho E., Kim H., Lee K. H., Kim E., Ye B. S. (2023). The development and evaluation of a self-efficacy enhancement program for older adults with mild cognitive impairment. Applied Nursing Research.

[B42-ejihpe-15-00226] Lee T. M., Chan F. H., Chu L. W., Kwok T. C., Lam L. C., Tam H. M., Woo J. (2017). Auditory-based cognitive training programme for attention and memory in older people at risk of progressive cognitive decline: A randomised controlled trial. Hong Kong Medical Journal = Xianggang Yi Xue Za Zhi.

[B43-ejihpe-15-00226] Li B.-Y., He N.-Y., Qiao Y., Xu H.-M., Lu Y.-Z., Cui P.-J., Ling H.-W., Yan F.-H., Tang H.-D., Chen S.-D. (2019). Computerized cognitive training for Chinese mild cognitive impairment patients: A neuropsychological and fMRI study. NeuroImage: Clinical.

[B44-ejihpe-15-00226] Li H., Su W., Dang H., Han K., Lu H., Yue S., Zhang H. (2022). Exercise training for mild cognitive impairment adults older than 60: A systematic review and meta-analysis. Journal of Alzheimer’s Disease.

[B45-ejihpe-15-00226] Liao Y. Y., Tseng H. Y., Lin Y. J., Wang C. J., Hsu W. C. (2020). Using virtual reality-based training to improve cognitive function, instrumental activities of daily living and neural efficiency in older adults with mild cognitive impairment. European Journal of Physical and Rehabilitation Medicine.

[B46-ejihpe-15-00226] Liu C.-C., Kanekiyo T., Xu H., Bu G. (2013). Apolipoprotein E and Alzheimer disease: Risk, mechanisms and therapy. Nature Reviews Neurology.

[B47-ejihpe-15-00226] Liu Y., Yu X., Han P., Chen X., Wang F., Lian X., Li J., Li R., Wang B., Xu C., Li J., Zheng Y., Zhang Z., Li M., Yu Y., Guo Q. (2022). Gender-specific prevalence and risk factors of mild cognitive impairment among older adults in Chongming, Shanghai, China. Frontiers in Aging Neuroscience.

[B48-ejihpe-15-00226] Longhurst J., Phan J., Chen E., Jackson S., Landers M. R. (2020). Physical therapy for gait, balance, and cognition in individuals with cognitive impairment: A retrospective analysis. Rehabilitation Research and Practice.

[B49-ejihpe-15-00226] Luo Y., Lin R., Yan Y., Li Y., Huang C., Chen M., Li H. (2024). Maintenance effects of short-period intensive creative expressive arts-based program (SPI-CrEAS) on cognitive function older adults with mild cognitive impairment: A pilot study. Geriatric Nursing.

[B50-ejihpe-15-00226] Malavera M., Silva F., García R., Rueda L., Carrillo S. (2014). Fundamentos y aplicaciones clínicas de la estimulación magnética transcraneal en neuropsiquiatría. Revista Colombiana de Psiquiatría.

[B51-ejihpe-15-00226] Molano J., Boeve B., Ferman T., Smith G., Parisi J., Dickson D., Knopman D., Graff-Radford N., Geda Y., Lucas J., Kantarci K., Shiung M., Jack C., Silber M., Pankratz V. S., Petersen R. (2010). Mild cognitive impairment associated with limbic and neocortical lewy body disease: A clinicopathological study. Brain.

[B52-ejihpe-15-00226] Montero-Odasso M., Zou G., Speechley M., Almeida Q. J., Liu-Ambrose T., Middleton L. E., Camicioli R., Bray N. W., Li K. Z. H., Fraser S., Pieruccini-Faria F., Berryman N., Lussier M., Shoemaker J. K., Son S., Bherer L. (2023). Effects of exercise alone or combined with cognitive training and vitamin d supplementation to improve cognition in adults with mild cognitive impairment: A randomized clinical trial. JAMA Network Open.

[B53-ejihpe-15-00226] Motter J. N., Rushia S. N., Qian M., Ndouli C., Nwosu A., Petrella J. R., Doraiswamy P. M., Goldberg T. E., Devanand D. P. (2024). Expectancy does not predict 18-month treatment outcomes with cognitive training in mild cognitive impairment. The Journal of Prevention of Alzheimer’s Disease.

[B54-ejihpe-15-00226] Moustaka K., Nega C., Beratis I. N. (2023). Exploring the impact of age of onset of mild cognitive impairment on the profile of cognitive and psychiatric symptoms. Geriatrics.

[B55-ejihpe-15-00226] Muhammad T., Govindu M., Srivastava S. (2021). Relationship between chewing tobacco, smoking, consuming alcohol and cognitive impairment among older adults in India: A cross-sectional study. BMC Geriatrics.

[B56-ejihpe-15-00226] Nakagawa S., Kowa H., Takagi Y., Kakei Y., Kagimura T., Sanada S., Nagai Y. (2024). Efficacy of a non-pharmaceutical multimodal intervention program in a group setting for patients with mild cognitive impairment: A single-arm interventional study with pre-post and external control analyses. Contemporary Clinical Trials Communications.

[B57-ejihpe-15-00226] Nwosu A., Qian M., Phillips J., Hellegers C. A., Rushia S., Sneed J., Petrella J. R., Goldberg T. E., Devanand D. P., Doraiswamy P. M. (2024). Computerized cognitive training in mild cognitive impairment: Findings in african americans and caucasians. Journal of Prevention of Alzheimer’s Disease.

[B58-ejihpe-15-00226] Orr M. E., Kotkowski E., Ramirez P., Bair-Kelps D., Liu Q., Brenner C., Schmidt M. S., Fox P. T., Larbi A., Tan C., Wong G., Gelfond J., Frost B., Espinoza S., Musi N., Powers B. (2024). A randomized placebo-controlled trial of nicotinamide riboside in older adults with mild cognitive impairment. GeroScience.

[B59-ejihpe-15-00226] Page M. J., McKenzie J. E., Bossuyt P. M., Boutron I., Hoffmann T. C., Mulrow C. D., Shamseer L., Tetzlaff J. M., Akl E. A., Brennan S. E., Chou R., Glanville J., Grimshaw J. M., Hróbjartsson A., Lalu M. M., Li T., Loder E. W., Mayo-Wilson E., McDonald S., Moher D. (2021). The PRISMA 2020 statement: An updated guideline for reporting systematic reviews. BMJ.

[B60-ejihpe-15-00226] Pantoni L., Poggesi A., Diciotti S., Valenti R., Orsolini S., Della Rocca E., Inzitari D., Mascalchi M., Salvadori E. (2017). Effect of attention training in mild cognitive impairment patients with subcortical vascular changes: The rehatt study. Journal of Alzheimer’s Disease.

[B61-ejihpe-15-00226] Park J.-H. (2022). Effects of virtual reality-based spatial cognitive training on hippocampal function of older adults with mild cognitive impairment. International Psychogeriatrics.

[B62-ejihpe-15-00226] Pavel A., Matei V., Paun R., Tudose C. (2023). How “subjective” is subjective cognitive decline?. Psychiatry and Clinical Psychopharmacology.

[B63-ejihpe-15-00226] Pellegrino L. D., Peters M. E., Lyketsos C. G., Marano C. M. (2013). Depression in cognitive impairment. Current Psychiatry Reports.

[B64-ejihpe-15-00226] Peng Z., Jiang H., Wang X., Huang K., Zuo Y., Wu X., Abdullah A. S., Yang L. (2019). The efficacy of cognitive training for elderly Chinese individuals with mild cognitive impairment. BioMed Research International.

[B65-ejihpe-15-00226] Petersen R. C. (2016). Mild cognitive impairment. CONTINUUM: Lifelong Learning in Neurology.

[B66-ejihpe-15-00226] Petersen R. C., Roberts R. O., Knopman D. S., Geda Y. E., Cha R. H., Pankratz V. S., Boeve B. F., Tangalos E. G., Ivnik R. J., Rocca W. A. (2010). Prevalence of mild cognitive impairment is higher in men: The mayo clinic study of aging. Neurology.

[B67-ejihpe-15-00226] Power R., Nolan J. M., Prado-Cabrero A., Coen R., Roche W., Power T., Howard A. N., Mulcahy R. (2020). Targeted nutritional intervention for patients with mild cognitive impairment: The cognitive impairment study (CARES) trial 1. Journal of Personalized Medicine.

[B68-ejihpe-15-00226] Prokopenko S. V., Bezdenezhnykh A. F., Mozheyko E. Y., Zubrickaya E. M. (2019). Effectiveness of computerized cognitive training in patients with poststroke cognitive impairments. Neuroscience and Behavioral Physiology.

[B69-ejihpe-15-00226] Raschetti R., Albanese E., Vanacore N., Maggini M. (2007). Cholinesterase inhibitors in mild cognitive impairment: A systematic review of randomised trials. PLoS Medicine.

[B70-ejihpe-15-00226] Rethlefsen M. L., Page M. J. (2021). PRISMA 2020 and PRISMA-S: Common questions on tracking records and the flow diagram. Journal of the Medical Library Association.

[B71-ejihpe-15-00226] Rotenberg S., Anderson N. D., Binns M. A., Skidmore E. R., Troyer A. K., Richardson J., Xie F., Nalder E., Bar Y., Davids-Brumer N., Bernick A., Dawson D. R. (2024). Effectiveness of a meta-cognitive group intervention for older adults with subjective cognitive decline or mild cognitive impairment: The ASPIRE randomized controlled trial. Journal of Prevention of Alzheimer’s Disease.

[B72-ejihpe-15-00226] Rovner B. W., Casten R. J., Hegel M. T., Leiby B. (2018). Preventing cognitive decline in black individuals with mild cognitive impairment: A randomized clinical trial. JAMA Neurology.

[B73-ejihpe-15-00226] Russo M. J., Kañevsky A., Leis A., Iturry M., Roncoroni M., Serrano C., Cristalli D., Ure J., Zuin D. (2020). Role of physical activity in preventing cognitive impairment and dementia in older adults: A systematic review. Neurologia Argentina.

[B74-ejihpe-15-00226] Saari T., Smith E. E., Ismail Z. (2021). Network analysis of impulse dyscontrol in mild cognitive impairment and subjective cognitive decline. International Psychogeriatrics.

[B75-ejihpe-15-00226] Sakurai T., Sugimoto T., Akatsu H., Doi T., Fujiwara Y., Hirakawa A., Kinoshita F., Kuzuya M., Lee S., Matsumoto N., Matsuo K., Michikawa M., Nakamura A., Ogawa S., Otsuka R., Sato K., Shimada H., Suzuki H., Suzuki H., Arai H. (2024). Japan-multimodal intervention trial for the prevention of dementia: A randomized controlled trial. Alzheimer’s and Dementia.

[B76-ejihpe-15-00226] Schneider L. S., Geffen Y., Rabinowitz J., Thomas R. G., Schmidt R., Ropele S., Weinstock M. (2019). Low-dose ladostigil for mild cognitive impairment: A phase 2 placebo-controlled clinical trial. Neurology.

[B77-ejihpe-15-00226] Shin H. Y., Kim H. R., Jahng G. H., Jin C., Kwon S., Cho S. Y., Park S. U., Jung W. S., Moon S. K., Ko C. N., Park J. M. (2021). Efficacy and safety of Kami-guibi-tang for mild cognitive impairment: A pilot, randomized, double-blind, placebo-controlled trial. BMC Complementary Medicine and Therapies.

[B78-ejihpe-15-00226] Steinbeisser K., Schwarzkopf L., Graessel E., Seidl H. (2020). Cost-effectiveness of a non-pharmacological treatment vs. “care as usual” in day care centers for community-dwelling older people with cognitive impairment: Results from the German randomized controlled DeTaMAKS-trial. The European Journal of Health Economics.

[B79-ejihpe-15-00226] Sung C. M., Jen H. J., Liu D., Kustanti C. Y., Chu H., Chen R., Lin H. C., Chang C. Y., Chou K. R. (2023a). The effect of cognitive training on domains of attention in older adults with mild cognitive impairment and mild dementia: A meta-analysis of randomised controlled trials. Journal of Global Health.

[B80-ejihpe-15-00226] Sung C. M., Lee T. Y., Chu H., Liu D., Lin H. C., Pien L. C., Jen H. J., Lai Y. J., Kang X. L., Chou K. R. (2023b). Efficacy of multi-domain cognitive function training on cognitive function, working memory, attention, and coordination in older adults with mild cognitive impairment and mild dementia: A one-year prospective randomised controlled trial. Journal of Global Health.

[B81-ejihpe-15-00226] Tan S. N., Tan C. (2021). Vortioxetine improves cognition in mild cognitive impairment. International Clinical Psychopharmacology.

[B82-ejihpe-15-00226] Wang P., Yang T., Peng W., Wang M., Chen X., Yang Y., Huang Y., Jiang Y., Wang F., Sun S., Ruan Y., Ding Y., Yao Y., Wang Y. (2024). Effects of a Multicomponent intervention with cognitive training and lifestyle guidance for older adults at risk of dementia: A randomized controlled trial. Journal of Clinical Psychiatry.

[B83-ejihpe-15-00226] Ware E. B., Higgins Tejera C., Wang H., Harris S., Fisher J. D., Bakulski K. M. (2024). Interplay of education and DNA methylation age on cognitive impairment: Insights from the health and retirement study. GeroScience.

[B84-ejihpe-15-00226] Wu J., He Y., Liang S., Liu Z., Huang J., Tao J., Chen L., Chan C. C. H., Lee T. M. C. (2023). Computerized cognitive training enhances episodic memory by down-modulating posterior cingulate-precuneus connectivity in older persons with mild cognitive impairment: A randomized controlled trial. American Journal of Geriatric Psychiatry.

[B85-ejihpe-15-00226] Xiao B., Fu L., Yang Z., Yu G. (2025). Effect of probiotics on cognitive function and cardiovascular risk factors in mild cognitive impairment and Alzheimer’s disease: An umbrella meta-analysis. Journal of Health, Population and Nutrition.

[B86-ejihpe-15-00226] Yan Y., Tian M., Wang T., Wang X., Wang Y., Shi J. (2023). Transcranial magnetic stimulation effects on cognitive enhancement in mild cognitive impairment and Alzheimer’s disease: A systematic review and meta-analysis. Frontiers in Neurology.

[B87-ejihpe-15-00226] Zhao J., Li H., Lin R., Xie M., Wang Y., Chen H. (2021). Effects of creative expression program on the event-related potential and task reaction time of elderly with mild cognitive impairment. International Journal of Nursing Sciences.

